# 

**Published:** 1851-05

**Authors:** 


### THE
### WESTERN JOURNAL
### OF
### MEDICINE AND SURGERY.

EDITED BY

LUNSFORD P. YANDELL, M. D.,

PROFESSOR OF PHYSIOLOGY AND PATHOLOGICAL ANATOMY IN THE UNIVERSITY OF LOUISVILLE,

AND

THEODORE S. BELL, M. D.

---

CXXXVII.—MAY, 1851.

---

THIRD SERIES.

Vol. VII......No. V.

---

LOUISVILLE, KY:

PUBLISHED MONTHLY BY PRENTICE & WEISSINGER,

PROPRIETORS AND PRINTERS.

1851.

